# The Effect of Elastic and Metallic Ligation Methods on the Unloading Forces for Three Different Types of Nickel-Titanium Archwires Inserted Into Metallic Brackets: An In-vitro Study

**DOI:** 10.7759/cureus.31952

**Published:** 2022-11-27

**Authors:** Odayy S Al-Horini, Feras Baba, Mohammad Y Hajeer, Mudar Mohammad Mousa

**Affiliations:** 1 Department of Orthodontics, University of Aleppo Faculty of Dentistry, Aleppo, SYR; 2 Department of Orthodontics, University of Damascus Faculty of Dentistry, Damascus, SYR

**Keywords:** austenite finish temperature, crystalline structure, stress induced martensite, metallic ligation, elastic logation, nickel-titanium wires, unloading foces, ligation methods

## Abstract

Introduction: One of the unique properties of the nickel-titanium (NiTi) archwires is the release of light continuous forces with a wide range of activation, which is affected by the method of ligating the wire to the bracket. Both elastomeric modules and metallic ligatures are the most common components to ligate the archwire to the bracket. This study aimed to evaluate the effect of elastomeric versus metallic ligatures on the released forces of three types of rectangular 0.016- by 0.022-inch NiTi wires.

Materials and Methods: This investigation evaluated three different situations of ligating the NiTi archwires to metallic brackets. The produced forces by these wires were evaluated using a modified three-point bending test (a universal testing machine: Testometric 350M^®^, Instron, Lincoln Close, Rochdale, England). A 0.022-inch slot-size premolar bracket was used for this investigation with zero torque and zero angulation (Master Series®, American Orthodontics, Sheboygan, USA). The first situation involved no ligation to the metallic brackets using three types of NiTi wires, whereas the second situation (the elastic ligation) involved the use of colored elastomeric modules with a 0.045-inch inner diameter and a 0.115-inch outer diameter (Color Ligatures^®^, American Orthodontics, Sheboygan, USA) to ligate the archwire into the metallic bracket's slot. In the third situation (metallic ligation), stainless steel metallic ligatures with a 0.012-inch (Preformed Ligature Wire^®^, American Orthodontics, Sheboygan, USA) were used to perform the ligation. The employed NiTi wires were 0.016 x 0.022-inch in diameter (American Orthodontics^®^, Sheboygan, Wisconsin, USA), and they were of three different types: (1) the superplastic wires (NT3-SE^®^), the thermally activated wires at 25°C (Thermal Ti-D^®^), and the thermally activated wires at 35°C (Thermal Ti-Lite^®^).

Results: The thermally activated wires at 35°C (Thermal Ti-Lite^®^) recorded the lowest force levels for the three ligation methods, while the highest result appeared with the superelastic type (NT3-SE^®^) and the thermally activated type at 25°C (Thermal Ti-D^®^) which showed a varied force for different ligation methods. Regarding the three different NiTi wires used, the elastic and metallic ligation increased the force levels for the superelastic type (NT3-SE^®^) over non-ligation by 50% and 110%, respectively, whereas the elastic ligation raised only the forces for the thermal types (Thermal Ti-D^®^ and Thermal Ti-Lite^®^) by 75% and 22%, respectively. Both thermally activated types (Thermal Ti-D^®^ and Thermal Ti-Lite^®^) released forces in the elastic ligation method greater than that of the metallic method by 125% and 20%, respectively.

Conclusion: The elastic ligation method raised the unloading forces in comparison with the non-ligation for all tested archwires, whereas the metallic ligation method raised the released forces for the superelastic type (NT3-SE^®^) and decreased those related to the activated types (Thermal Ti-D^®^, Thermal Ti-Lite^®^) due to the increased friction and the instability of the crystalline structure. Only the heat-activated type at 35°C (Thermal Ti-Lite^®^) reflected the unique properties of the NiTi wires in producing light continuous forces with a wide range of activations regardless of the type of ligation*.*

## Introduction

The nickel-titanium archwires are considered the most attractive wires to orthodontists during the first stage of orthodontic treatment owing to their unique property of releasing light continuous forces with a wide range of activation [1]. This property could be affected by two major factors; the first one is the generation of NiTi wires and their diameter [2-4], while the second factor is the method of ligating the wire to the bracket [5,6]. Elastomeric modules and short metal ligatures are the most common components used to ligate the archwires to the bracket [7].

The advantages of elastomeric modules include reducing the sitting time of the patient in the dental chair and being an easy and fast procedure to replace, while the disadvantages include the accumulation of dental plaque around brackets, especially in non-cooperative patients [8]; these elastic rings are usually available in different colors and different diameters depending on bracket dimensions [9]. On the other hand, the short metallic ligatures, the oldest method to ligate wire to the bracket, depend on making metal rings around the bracket to firmly hold the archwire. The metal wire, which is used in this method, is made from stainless steel with a small diameter. This method is considered better than elastic ligation owing to the possibility of the accumulation of dental plaque [10]. In most cases, during the leveling and alignment stage, the teeth will not be in the same plane, and the engaging of the archwire to the bracket will produce forces on the teeth; those forces will move the teeth to the desired position [11]. So the effect of the method used at the junction between the archwire and bracket is very important to understand the biological reaction of that force and to exclude methods that do not affect forces properly.

In light of some in vitro studies, only a few authors focused on the effect of ligation methods on the force level during leveling and alignment of teeth. Kasuya et al. [12] evaluated the effect of elastic ligation versus metallic ligation on 0.016-inch round NiTi wires and concluded that metallic ligation was better than elastic ligation in terms of allowing the superelastic properties to be expressed. Henriques et al. [13] compared the force released from different brands of 0.016-inch NiTi wires via elastic and metallic ligation and discovered that metallic ligation increased the released forces across all wire brands. Higa et al. [14] evaluated the deflection forces using conventional and thermally activated NiTi archwires with small diameters (i.e., 0.014- and 0.016-inch archwires). These were tied by elastic and metallic ligatures showed that in large deflections, the conventional NiTi archwires released higher forces than the thermally activated NiTi archwires, whereas the forces were randomly dissimilar for all tested archwires when elastomeric ligation was used.

Several previous studies [12-14] evaluated the effect of different ligation methods on the force levels of NiTi initial archwires, whereas studies on rectangular NiTi leveling and alignment archwires were mostly confined to friction analysis than force level assessment. Leite et al. [15] compared the frictional forces for 0.016x0.021 conventional NiTi wires and found that the metal ligature raised the friction forces over elastomeric ligating. In contrast, Matarese et al. [16] concluded that no significant differences were found between elastomeric and metallic ligation in terms of friction forces of 0.016X0.022 conventional NiTi wires.

Therefore, the current study aimed to evaluate the effect of elastomeric ligating versus metallic ligating on the released forces of three types of rectangular 0.016- by 0.022-inch NiTi wires: the superelastic wire, the thermal heat-activated wires at 25°C and 35°C activation temperatures. The null hypothesis: There were no differences between elastomeric and metallic ligation in terms of the produced orthodontic forces when using the three different types of NiTi archwires.

## Materials and methods

Study design and settings

This study was a single-blind randomized controlled in-vitro study for evaluating the effect of two different ligation methods in the released forces of three different types of NiTi archwires. The Department of Orthodontics at Aleppo Dental School, Aleppo University, Syria, and the Faculty of Mechanical Engineering at Aleppo University, Syria, collaborated on this project.

Sample size calculation

The sample size was calculated by using Minitab® Version 17 (Minitab Inc., State College, Pennsylvania, USA) depending on the study of Kasuya et al. [12], which evaluated the effect of different types of ligation on superelastic NiTi wires at a sample size of five wires for each ligation method, whereas the standard deviation was (0.22) while the main difference of the load forces between different ligation methods was (+1.45N) at confidence level 95%, six wires were found to be required for each group.

The wires used in this experiment

The types of nickel-titanium wires that were used in the experiment included: superplastic NiTi wires with moderate force levels (NT3-SE®, American Orthodontics, Sheboygan, Wisconsin, USA), thermal NiTi wires activated over 25° with moderate force levels (Thermal Ti-D®, American Orthodontics, Sheboygan, Wisconsin, USA), and thermal NiTi wires activated over 35° with light forces (Thermal Ti-Lite®, American Orthodontics, Sheboygan, Wisconsin, USA). All wires with dimensions of 0.016 x 0.022-inch; the length of tested samples was 30 mm, cut from the posterior section of the arch by a heavy straight cutter, while the brackets were Roth 0.022 premolar brackets with zero torque and zero angulations (Master Series®, American Orthodontics, Sheboygan, USA).

The experimental groups

**Figure 1:**
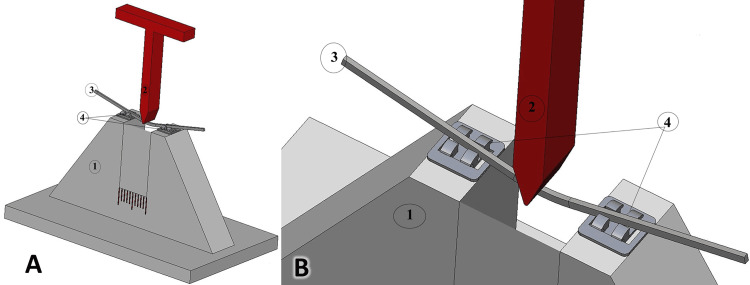
Simulation of the wire and the two metallic brackets in case of no ligation. A: An overview of the whole assembly. B: A close-up view of the experimental test. 1: Plexiglas bracket holder; 2: The crosshead used in this experiment; 3: The Nickel-Titanium (NiTi) archwire; 4: the two metallic brackets

The first group (no ligation) included three different types of nickel-titanium wires inserted into two brackets without any type of ligation (Figure 1). The second group (elastic ligation) consisted of three different types of nickel-titanium wires that were ligated to two brackets by colored ligature elastomeric rings with a 0.045-inch inner diameter and 0.115-inch outer diameter (Color Ligatures®, American Orthodontics, Sheboygan, USA). The third group (metallic ligation) included three different types of nickel-titanium wires ligated to two brackets by a stainless-steel metal ligature with a 0.012-inch (0.25mm) diameter (Preformed Ligature Wire®, American Orthodontics, Sheboygan, USA).

Outcome measures: load/deflection curves

**Figure 2:**
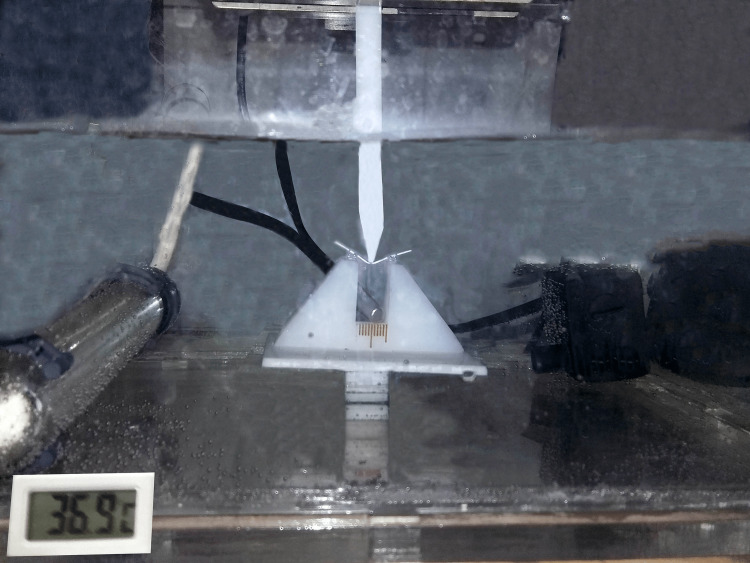
The water bath used in this study with the tested specimen in place. The temperature of the water bath was stabilized at approximately 37°C.

A modified three-point bending test was performed by using a universal testing machine (Testometric 350M®, Instron, Lincoln Close, Rochdale, England) with a modified load cell of 100N, a water bath made from Plexiglas to simulate the clinical situation by adding two premolar brackets Roth 0.022 (Master Series®, American Orthodontics, Sheboygan, USA) with 0 torque and 0 angulations to the base of it, and a water heater for stabling the temperature at 37°C [4,13,14] as shown in Figure 2. The length of the tested wires was 30 mm, while the spam length was 10 mm. The crosshead deflected each wire by 3.1 mm with a 1 mm/min speed rate and made point contact with the center of the tested wire. The deflection was done in the horizontal direction (buccal-palatal). The setting of this experiment was done following the guidelines of ISO/DIS 15841 [17]. The resulting load/deflection curves were analyzed using Solid Works® version 2012 software (SolidWorks®, Dassaultsystems, Concord, Massachusetts, USA) and Microsoft Excel (Microsoft Excel™ 2013, Microsoft Corporation, Redmond, Washington, USA). The unloading forces were evaluated at two points: 0.5 and 1 mm. These points represented the wire deflection and also the forces applied to the tooth at the beginning (i.e., 1mm) and the end (i.e., 0.5 mm) of the unloading stage.

Statistical analysis

The statistical analysis was performed using the IBM Corp. Released 2011. IBM SPSS Statistics for Windows, Version 20.0. Armonk, NY: IBM Corp. To assess the normality of the distributions, Kolmogorov-Smirnov tests were used. For the analysis of variance, one-way ANOVA tests were employed, and post-hoc Sidak's tests, with a level of significance set at 5%, were used to find significant differences between each pair of groups.

The error in the method

Three different wires from the tested sample were chosen randomly, with the concern being one wire from each different group to repeat the load/deflection test; then, the reliability test (Cronbach’s alpha test) was performed to check the reliability of the method. The results showed a high value of alpha Cronbach (0.984), indicating the method's reliability.

## Results

The effect of wire type

**Figure 3:**
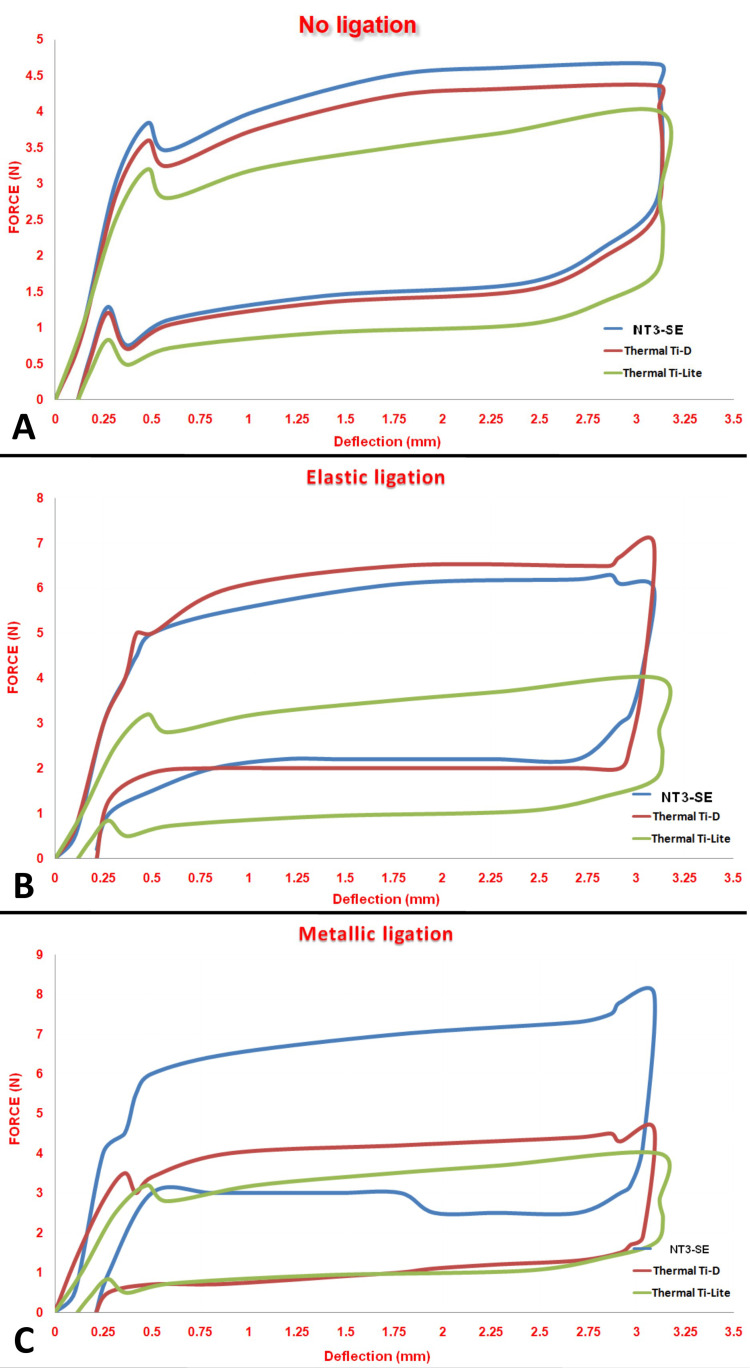
Load/deflection curves for the three types of NiTi wires when evaluating the three different ligation situations. A: No ligation; B: Elastic ligation; C: Metallic ligation.

All tested wires showed a classic load/deflection curve of NiTi alloys with a flattening plateau during loading and unloading for the no-ligation group, while a change was noticed for the Thermal Ti-D® wires in the elastic ligation group and NT3-SE® in both the elastic and metallic ligation groups. That change involved an increase in loading and unloading values, a variation in the forces between the start and the end of the plateau, and a decrease in the liner part of the unloading curve (Figure 3).

The NT3-SE® and Thermal Ti-D® delivered equal forces, while Thermal Ti-Lite® delivered lower forces for the unloading points (0.5-1mm) in the no-ligation and elastic ligation groups. On the other hand, the Thermal Ti-D® and Thermal Ti-Lite® delivered almost equal forces, while NT3-SE® delivered higher forces for the unloading points (0.5-1mm) in the metallic ligation group (Table 1).

**Table 1:** Descriptive statistics of the unloading forces for the three types of wires according to the different ligation methods SD: standard deviation; Max: maximum value; Min: minimum value.

Ligation methods	Unloading value	Wire type											
		NT3-SE^®^				Thermal Ti-D^®^				Thermal Ti-Lite^®^			
		Mean	SD	Max	Min	Mean	SD	Max	Min	Mean	SD	Max	Min
No ligation	0.5 mm	0.95	0.18	1.20	0.70	1.05	0.18	0.80	1.30	0.71	0.20	0.99	0.44
	1 mm	1.25	0.18	1.5	1.00	1.35	0.18	1.60	1.10	0.83	0.19	1.11	0.58
Elastic ligation	0.5 mm	1.5	0.18	1.8	1.30	1.80	0.23	2.20	1.60	0.70	0.18	0.94	0.44
	1 mm	1.85	0.18	2.10	1.60	1.90	0.19	2.15	1.64	1.05	0.18	1.30	0.80
Metallic ligation	0.5 mm	3.00	0.17	3.25	2.80	0.45	0.18	0.70	0.20	0.55	0.18	0.80	0.30
	1 mm	3.2	0.187	3.50	3.00	0.90	0.18	1.10	0.60	0.85	0.18	1.10	0.60

The statistical evaluation reveals that there was a significant difference between the thermally activated type at 35°C (Thermal Ti-Lite®) and the other types (p < 0.01) for both the no-ligation and elastic ligation groups, while for the metallic ligation group, the significant difference was found between the two thermal activated types and the superelastic type (NT3-SE®) for both unloading points (0.5-1mm; p < 0.01; Table 2).

**Table 2:** Post-hoc tests for the pairwise comparisons between the ligation methods for the three types of archwires.

Ligation method	Unloading value	Pairwise comparisons	P-value
No ligation	0.5 mm	NT3-SE vs. Thermal Ti-D	0.767
		NT3-SE vs. Thermal Ti-light	0.150
		Thermal Ti-D vs. Thermal Ti-light	0.027
	1 mm	NT3-SE vs. Thermal Ti-D	0.759
		NT3-SE vs. Thermal Ti-light	0.006
		Thermal Ti-D vs. Thermal Ti-light	<0.001
The elastic ligation	0.5 mm	NT3-SE vs. Thermal Ti-D	0.037
		NT3-SE vs. Thermal Ti-light	<0.001
		Thermal Ti-D vs. Thermal Ti-light	<0.001
	1 mm	NT3-SE vs. Thermal Ti-D	0.975
		NT3-SE vs. Thermal Ti-light	<0.001
		Thermal Ti-D vs. Thermal Ti-light	<0.001
The metallic ligation	0.5 mm	NT3-SE vs. Thermal Ti-D	<0.001
		NT3-SE vs. Thermal Ti-light	<0.001
		Thermal Ti-D vs. Thermal Ti-light	0.736
	1 mm	NT3-SE vs. Thermal Ti-D	<0.001
		NT3-SE vs. Thermal Ti-light	<0.001
		Thermal Ti-D vs. Thermal Ti-light	1.000

The effect of the ligation method

**Figure 4:**
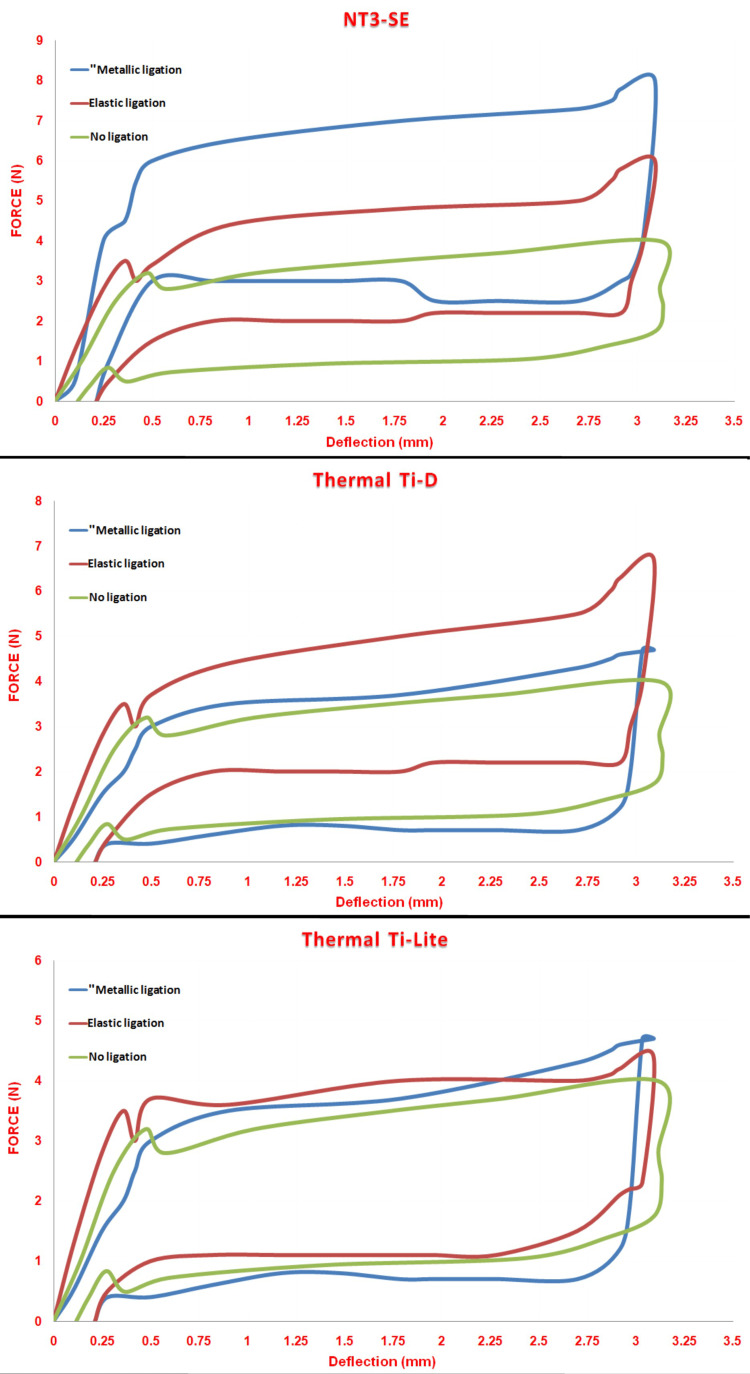
Load/deflection curves for the different ligation methods when evaluating the three types of Nickle-Titanium (NiTi) archwires. A: The superelastic NiTi wire (NT3-SE); B: Thermally activated NiTi wire at 25°C (Thermal Ti-D®); C: Thermally activated NiTi wire at 35°C (Thermal Ti-Lite®).

The use of elastic ligation raised the release force compared with no-ligation by 40% to 60% for the NT3-SE®wires, by 55% to 71% for Thermal Ti-D® wires, and by 22% for Thermal Ti-Lite® wires, respectively. The use of metallic ligation raised the forces by 110% to 120% for the NT3-SE® wires, while the forces declined by 44% for Thermal Ti-D® wires and by 16% for Thermal Ti-Lite® wires (Figure 4).

On the other hand, the forces were greater in the metallic ligation group than the elastic one by 40% to 70% for the NT3-SE®. At the same time, the forces were slightly greater in the elastic ligation group than those of the metallic group by 20% for the Thermal Ti-Lite® wires and by 120% for the Thermal Ti-D® wires, respectively, as shown in the first table. Significant differences were observed when comparing the three ligation methods for the NT3-SE® and Thermal Ti-D® types. No significant difference was detected for the wire type Thermal Ti-Lite® (Table 3).

**Table 3:** Post-hoc tests for pairwise comparisons between the three types of Nickle-Titanium archwires and the three ligation situations.

Wire type	Unloading value	Pairwise comparisons	P-value
NT3-SE®	0.5 mm	No ligation vs. elastic ligation	<0.001
		No ligation vs. metallic ligation	<0.001
		Elastic ligation vs. metallic ligation	<0.001
	1 mm	No ligation vs. elastic ligation	<0.001
		No ligation vs. metallic ligation	<0.001
		Elastic ligation vs. metallic ligation	<0.001
Thermal Ti-D®	0.5 mm	No ligation vs. elastic ligation	<0.001
		No ligation vs. metallic ligation	<0.001
		Elastic ligation vs. metallic ligation	<0.001
	1 mm	No ligation vs. elastic ligation	<0.001
		No ligation vs. metallic ligation	<0.001
		Elastic ligation vs. metallic ligation	<0.001
Thermal Ti-Lite®	0.5 mm	No ligation vs. elastic ligation	0.995
		No ligation vs. metallic ligation	0.408
		Elastic ligation vs. metallic ligation	0.542
	1 mm	No ligation vs. elastic ligation	0.199
		No ligation vs. metallic ligation	0.999
		Elastic ligation vs. metallic ligation	0.244

## Discussion

The modern orthodontic NiTi wires could be classified into the types of active austenite and active martensite, i.e., super elastic and heat activated, respectively [1]. Both types show martensitic transformation, which happens under different stresses, such as superelastic wires, or temperature variation, such as heat-activated wires [2,3]. The austenite structure is stiffer than the martensitic [3,8,18], so when the NiTi wires are bent in the austenite phase, they will produce higher forces than when bent in the martensitic phase. In this sense, heat-activated types typically exhibit less force than superelastic types because of their differences in crystalline structures, formation conditions, and metal compositions [4,8].

Bending a NiTi wire at a constant temperature will produce a dramatic transformation of the internal structure. It is mainly affected by two major factors. The first is the temperature [1-4,8,19,20]. In the case of high temperature than the austenite finish temperature, the wire is in the austenite structure, and the martensite transformation occurs solely due to applied stress [2,3,8,20]. In contrast, if the temperature is lower than the austenite finish temperature, the transformation will occur as compensation for temperature and applied stress. The second factor is stress introduced martensite (SIM) phase, which is affected by four factors: geometry, ligation methods, friction, and sliding [4-7,9,10,11].

The current study applied a modified bending test to three NiTi orthodontic wires. One was super elastic (active austenite), while the others were heat-activated (active martensite) but employed different activation degrees. All the wires were manufactured by the same manufacturer with the same dimensions (0.016 x 0.022-inch). The modified bending test was performed by two 0.022-inch Roth brackets adhesive to the wire holder while three different ligation methods were used; no ligation, elastic ligation, and metallic ligation, at a constant temperature of 37 centigrade degrees; the load/deflection curve for each combination of wire/ligation method was evaluated to determine the force levels at two unloading points (0.5-1 mm).

The combination of wire/no ligation

There was no ligation between the wire and the brackets in this combination, so it was anticipated that the wire can slide and deflect freely through the brackets without any pressure or friction. The load/deflection curve for the three types of wires showed a classic load/deflection curve of NiTi alloys with a flattening plateau during the loading and unloading stages. That means the wire was free to move through the brackets, and no frictional or mechanical effects were observed.

For unloading points of 0.5mm, no significant differences in force levels were detected among all wire types, which agrees with previous reports showing no differences in force levels between superelastic and heat-activated NiTi wires for small deflections [13,20]. While for unloading points of 1 mm, no difference was detected between the superelastic type (NT3-SE®) and the thermally activated type at 25°C (Thermal Ti-D®), whereas a significant difference was detected between the previous types and the thermal type activated at 35°C (Thermal Ti-Lite®). This difference could be explained by the relationship between the austinite finish temperature and the wire stiffness. According to our previous research regarding the phase structure and the austinite finish (Af) temperatures of these wires, we determined the Af temperature for the superelastic type (NT3-SE®) is at 16.84°C and for the heat activated at 25°C (Thermal Ti-D®) at 23°C, while the heat activated at 35°C (Thermal Ti-Lite®) at 33.99 °C. That agrees with the claim that NiTi wires with a lower Af temperature will be stiffer than wires with a higher one [20].

The combination of wire/elastic ligation

An elastic ligation (elastomeric module) was applied around brackets in this combination. The elastic modules that engage the wire into the bracket during orthodontic treatment play an important role in moving the teeth into the desired positions [5,10]. From the mechanical point of view, the wires act like a free span, but when the elastic ring is used, the wire will act as an attached span [5,19], so the mechanical behavior of the wires will change. Regarding the load/deflection curve for the tested NiTi wires, changes appeared for two types (NT3-SE®, Thermal Ti-D®), while Thermal Ti-Lite® almost had the same characteristics.

The detected changes at the load/deflection curve could be summarized as an increase in loading and unloading forces and hystereses (the variation between loading and unloading forces). Proffit et al. [19] claim that changing the span attached would double the wire strength but reduce its range to half. However, that claim did not mention the effect of the differences in wire materials.

On the other hand, no change was noticed for the thermal wire activated at 35°C (Thermal Ti-Lite®). That means this type of wire does not follow the previous claim. This may be attributed to the unsteady crystalline structure of this wire, which in its turn affected the force levels that were needed to start the martensitic transformation under stress [18].

For the two unloading points (0.5 and 1 mm), no significant differences in force levels were detected between the super elastic type (NT3-SE®) and the heat activated at 25°C (Thermal Ti-D®), while the thermal heat activated at 35°C had significant differences compared to the other wires. That would be a clear result of the explanations given before.

The combination of wire/metallic ligation

In this combination, a thin metal wire diameter of 0.25 mm was used to ligate the wires to the brackets. This method was the original way employed to hold the wire into brackets [5,10]. As was explained for elastic ligating, ligating the wires by metal ligature would affect the mechanical behavior, which in turn would affect the load/deflection curve and the force levels of the wires. Regarding the load/deflection curve for the tested NiTi wires, only the super elastic type (NT3-SE®) showed a change, while for both thermal types (Thermal Ti-D®, Thermal Ti-Lite®), almost no change was detected.

As in elastic ligation, the detected changes in the load/deflection curve for the superelastic wire (NT3-SE®) with metal ligating could be summarized as an increase in both loading and unloading forces as well as an increase in hystereses (the variation between loading and unloading forces). Also, as previously-explained, ligating the span will change its strength, stiffness, and range, which will be manifested as greater loading and unloading forces.

In contrast, both thermal wires (Thermal Ti-D®, Thermal Ti-Lite®) did not show any sign of change in the characteristics of the load/deflection curve, and almost the released forces were equal. This means that metal ligating played a role in balancing the released forces between both heat-activated wire types. Even though they were not equal in the no-ligation situation, it is estimated that the prestress that was applied from the metal ligature contributed to moving the crystalline structure toward the martensite phase, thereby reducing the threshold of stress martensite transition [19]. Thus, both wires had an unstable crystalline structure, resulting in released equal forces and reducing the expected effect of metal ligation to nil. For the two unloading points (0.5-1) mm, no significant differences in force levels were detected between heat-activated wires (Thermal Ti-D®, Thermal Ti-Lite®), while the superelastic wire (NT3-SE®) had significant differences with both the previous wires.

The effect of the ligation method on the superelastic wire (NT3-SE®)

Comparing the forces of the superelastic type in the three ligation situations (no ligation, elastic ligation, and metallic ligation) could be the easiest way to determine how each method affects the wire's behavior. Theoretically, in the no-ligation situation, the wire will bend and recover freely in the absence of any external factors [5,19]. In other words, the wire demonstrates pure behavior that consists of wire geometric shape effect and wire material properties. In contrast, with any ligation (elastic or metallic), the mechanical behavior will be more complex. In contrast, another factor, i.e., arch wire status will affect the reaction, not only the properties of wire materials, Profit et al. [19] demonstrated that changing the archwire status from unligated to ligated (attached span) increases stiffness by four times, increases strength twice, and decreases range and springiness. Observing the force levels resulting from no ligation and elastic ligation, we noticed an increasing amount of force between 40% and 60% and between 110% and 120% for metallic ligation. That may be the mechanical expression of the increasing strength and stiffness. But between the metallic and the elastic ligations, the forces are raised for metallic by 40% to 70%. This implies the tight metallic ligation doubles the strength and stiffness of the wires over the relatively loose elastic ligatures. This result agrees with the results of Kasuya et al. [12], Henriques et al. [13], and Higa et al. [14], who concluded that wires ligated with metallic ligature delivered higher forces than elastic or no ligature at all.

The effect of the ligation method on the heat-activated wire at 25 °C (Thermal Ti-D®)

According to our previous experiment with heat-activated wires at 25°C (Thermal Ti-D®), those wires had moderate forces compared to the other types and a stable crystalline structure at room temperature [20]. Regarding the force levels for this wire type through different ligation methods, this type of wire showed unique properties. When no ligation was used, the wire delivered a moderate force due to the effect of bending and recovery without any external factor, whereas when elastic ligation was used, it showed an increase in loading and unloading forces of between 55% and 75% over the no ligation situation, which could be explained by the change in strength and stiffness of wires due to the ligation situation, which was carefully described in the previous section.

In contrast, the metallic ligation, instead of increasing the force levels, depressed them by 44%. When a ligature wire is placed around the brackets, it will usually hold the wire rigidly in the brackets, so minimum sliding or slipping of the wire and maximum amount of friction will take place [15,16]. Another point is the pre-stress that is applied to wires [19], which plays a role in pushing the crystalline structure to be more martensitic (more ductility). This way, the released force will be less, and the threshold to start the martensitic transition will be reduced. In our viewpoint, the metallic ligature, when used with this type, saves the unique properties of this wire (light forces) at the expense of increased friction forces and stops the wire from slipping through brackets.

When comparing the force levels between elastic and metallic ligation, the forces were higher for elastic ligation by 125%, which clearly shows how tight ligating reduces the release force at the cost of increasing friction and limiting wire slipping.

The effect of the ligation method on the heat-activated wire at 35°C (Thermal Ti-Lite®)

The findings of heat-activated wires at 35°C (Thermal Ti-Lite®) had the most variable values of the three tested types. According to our previous experiment, Thermal Ti-Lite® wires exerted light forces compared to the other types and a combined crystalline structure [20]. With the varying ligation methods, the force levels for this wire type showed specific properties. When no ligation was used, the wire delivered a light force due to the effect of bending and recovery without any external factor, whereas with elastic ligation, it showed an increase in loading and unloading forces for a 1 mm defection point by 22% over the former situation. It is to be noted that the forces were equal for the point of 0.5 mm. That result agrees somewhat with the findings of Higa et al. [14], who noticed the significant effect of ligation methods only in large deflection. Similarly, Profit et al. [19] attributed the differences to the strength and stiffness changes in attached wires. This idea has been elaborated in the previous section. While for metallic ligation, the forces were lower by 16% for deflection point 0.5 mm than for non-ligation, although it was equal for the point 1 mm. Further, the metallic ligation was also lower by 20% than the elastic ligation. The decline in the forces was also explained clearly and attributed to the increasing friction of wires through rigid ligation. Additionally, no significant difference was detected for this type of wire in all three ligation situations. In other words, the effect of ligation methods on this type of wire was almost absent, and this wire type held the unique NiTi wire properties of releasing light continuous forces with a wide range of activation.

Limitations

One limitation of the current study is the dependence on archwires manufactured by a single company. Therefore, in future studies, an unbiased and reliable assessment of different archwires from different manufacturers is mandatory. The second limitation is the mechanical testing method used in the current work. We used an Instron-350M device for the bending test, which was not originally designed to measure light forces. Therefore, the load cell was adjusted to measure light force levels; this may slightly raise concerns about the precision of measured values.

## Conclusions

The elastic ligation method raises the unloading forces compared to the non-ligation method for all tested archwires, with varying percentages of increase. The metallic ligation raised the unloading forces greater than that of the elastic ligation for the super-elastic type (NT3-SE®) only, whereas, for both the heat-activated types (Thermal Ti-D® and Thermal Ti-Lite®), the forces were reduced due to an increase in friction and the instability of the crystalline structure.

The heat-activated type at 35°C (Thermal Ti-Lite®) showed the minimum effect of changing the ligation methods, followed by the heat-activated type at 25°C (Thermal Ti-D®), whereas the superelastic type (NT3-SE®) had the maximum effect in terms of changes in the loading forces. The super-elastic type (NT3-SE®) exhibited a mechanical behavior similar to that of the stainless steel archwire whether a tight or loose ligation method was used, whereas the heat-activated type at 25°C (Thermal Ti-D®) demonstrated a mechanical behavior similar to that of a stainless steel archwire when elastic ligation was used and a NiTi archwire when a metallic ligation was used. Only the heat-activated type at 35°C (Thermal Ti-Lite®) reflected the unique properties of the NiTi wires in producing light continuous forces with a wide range of activations regardless of the type of ligation.
